# Compassion fatigue in helping professions: a scoping literature review

**DOI:** 10.1186/s40359-024-01869-5

**Published:** 2025-04-08

**Authors:** Amelia Mohd Noor, Dodi Suryana, Engku Mardiah Engku Kamarudin, Noor Banu Mahadir Naidu, Siti Rozaina Kamsani, Priyalatha Govindasamy

**Affiliations:** 1 Department of Guidance and Counseling, Universiti Pendidikan Sultan Idris, Tanjung Salim, Perak Malaysia; 2 Program Study of Guidance and Counseling, Universitas Pendidikan Indonesia, Bandung, Jawa Barat Indonesia; 3 Department of Counselor Education and Counselling Psychology, Universiti Putra Malaysia, Serdang, Selangor Malaysia; 4 Department of Moral, Civic Studies and Character Development, Universiti Pendidikan Sultan Idris, Tanjung Salim, Perak Malaysia; 5 Department of Psychology and Counselling School of Applied Psychology, Universiti Utara Malaysia, Sintok, Kedah Malaysia; 6 Department of Psychology, Universiti Pendidikan Sultan Idris, Tanjung Salim, Perak Malaysia

**Keywords:** Fatigue, Conceptual analyses, Helping professions, Predictor, Scoping literature review

## Abstract

**Background:**

Generalizing the concept of compassion fatigue across healthcare settings or professions is difficult because compassion fatigue is a complex and abstract concept. Compassion fatigue is described as a result in the form of behaviors and emotions resulting from learning of another person's traumatic event. Compassion fatigue is considered a 'cost of caring.' This study was a scoping literature review that aimed to identify what is known about compassion fatigue in helping professions.

**Methods:**

A systematic search was conducted on electronic databases, namely ScienceDirect, PubMed, and Taylor and Francis. Data analysis was conducted using PRISMA-ScR (Preferred Reporting Items for Systematic Reviews and Meta-Analyses). Study results were mapped based on the following criteria: 1) conceptual analysis; 2) predictor factors; and 3) research progress. A total of 43 articles met the inclusion and eligibility criteria for further review in this scoping literature review.

**Results:**

The results showed that it is difficult to imagine how a conceptual model of compassion fatigue could be equally relevant and applicable to various helping professions. Factors that can influence compassion fatigue are divided into personal factors (professional factors and sociodemographic factors), such as resilience, burnout, moral courage, emotional control, mindfulness, work experience, professional competence, and professional efficacy, and work-related factors such as traumatic experiences, life disorders, number of patients treated, job satisfaction, emotional support, social support, and fluctuations in interactions with suffering patients. Research on compassion fatigue has developed a lot, especially in the health sector, especially nursing using experimental, cross-sectional, and literature review research methods.

**Conclusion:**

Further analysis is needed in developing a conceptual analysis of compassion fatigue that focuses on other fields of work more specifically and comprehensively by paying attention to, aspects, determinants, and validity of compassion fatigue symptoms.

## Introduction

The issue of compassion fatigue is often overlooked in the guidance and counseling education environment. Many academics and practitioners have comprehensively reviewed the concept of compassion fatigue in various other helping professions, such as doctors, psychotherapists, psychologists, social workers, paramedics, counselors, teachers, and others [1]. From the research findings of the last 20 years, awareness of the negative impact of compassion fatigue and the importance of maintaining quality of life in helping professions has been widely studied. Compassion fatigue is a complex and abstract concept [2]. Over the years, the term compassion fatigue has often been equated or replaced with the terms Secondary Traumatic Disorder (STD) and Burnout [3]. The term compassion fatigue was then first used in the health sector in 1992 by Joinson and was defined as ‘loss of the ability to nurture’, namely the loss of ability to nurture in emergency nurses. However, when examined from the perspective of education, psychology and counselling, Compassion can be defined as a feeling that arises when witnessing the suffering of others and motivates the desire to help [4]. Compassion is also conceptualised as a cognitive, affective and behavioural process that includes five elements that refer to the self and others, namely: 1) Recognising suffering; 2) Understanding the totality of suffering in the human experience; 3) Feeling empathy for the person suffering and relating to the distress (emotional resonance); 4) Tolerating uncomfortable feelings that arise in response to the person suffering (e.g. distress, anger, fear) so as to remain open and accepting of the person suffering; and 5) Motivation to act to alleviate suffering [5].

In the context of cultures in Europe and other countries except ASEAN, including Indonesia and Malaysia, exploring the essence of defining a comprehensive concept of compassion fatigue is important, considering that the literature review mostly examines the concept of compassion fatigue related to one of the helping professions, namely the nursing field. In fact, other occupations related to helping traumatised people can also carry the risk of experiencing pain as a direct reaction to exposure to traumatic events experienced by others. This difference is essential for follow-up, that in 15 years of experience, represents Compassion fatigue is often experienced by helping professionals such as nurses, social workers, psychotherapists, and other professions that often have demands to provide high levels of care to clients [6]. Helping professions are defined as those that involve professional interactions between an expert and a client, fostering growth or addressing a person's physical, psychological, intellectual or emotional condition, through medical treatment, nursing, psychotherapy, psychological counselling, social work, education or coaching [7, 8].

Researchers such as Alshammari & Alboliteeh, Portoghese et al.,, and Timofeiov-Tudose & Măirean, 2023 argue that [9–11] argues that compassion fatigue leads to high levels of burnout when seeing patients suffer and has negative consequences for caring that can impact the entire organization. The increasing awareness of the importance of studying work-related stress faced by healthcare workers has led some researchers to view the term 'compassion fatigue' as problematic and vague [2, 12]. For two decades, the term compassion fatigue has brought considerable attention to the nursing profession and health care workers [13]. Research results [14] that the issue of compassion fatigue of individuals who are highly compassionate will have consequences for themselves and cause harm in many situations. Under these conditions, when individuals attempt to see things from the perspective of the person who is suffering, then the individual may also suffer This situation is called compassion fatigue, which is an unintended consequence of work related to people who suffer [3].

Although awareness of compassion fatigue in education has emerged, research exploring the study of compassion fatigue is still limited. Most of the research on compassion fatigue was conducted in the Americas [15–18]. There are also compassion fatigue studies in other continents, namely Asia [19, 20], European [21], and Middle East [22]. Studies on compassion fatigue in education, especially in Southeast Asia, are scarce. The decision to use the scoping literature review method in this study was based on the observation that many articles deemed relevant to the compassion fatigue review only focus on the nursing profession and workers in the health sector.

The impact of this research not being explored, researchers will be comprehensively disorientated from the unclear definition of compassion fatique philosophically, compassion fatique. will not even know the pattern of emerging research methods, and inaccuracies to find research gaps at the basic, applied, as well as developmental research levels. Another impact will be the confusion for policy makers to make decisions related to curriculum, resources, and regulations. besides the absence of a platform built on the analysis of theoretical and empirical data to support strategy design, implementation in educational settings.

To fill the gap and serve as a foundation for more systematic research on compassion fatigue, this study aims to identify what is known about compassion fatigue in helping professions such as nurses, social workers, psychotherapists, counsellors, psychologists and other helping professions, which includes the conceptualization, predictors, and research development of compassion fatigue in helping professions.

### Methodology

This research uses a qualitative approach with the Scoping Literature Review method. Literature Review is a systematic, explicit, and usable method for identifying, evaluating, and synthesizing a collection of works on a topic produced by researchers, academics, and practitioners [23]. A scoping review is a type of review that aims to extract as much relevant data from the literature as possible to provide a complete picture of what has been done [24]. Scoping reviews can identify key concepts, the size of the research pool, the type of evidence available, and research gaps [25].

The steps in conducting a scoping review follow the guidelines developed by Arksey & O’Malley [24], which consists of five stages, including: 1. identifying the research question 2. identifying relevant studies 3. selecting relevant studies 4. mapping the data 5. compiling, summarizing and reporting the results.

#### Stage 1. Identifying the research question

This study aims to identify what is known about compassion fatigue in helping professions. To achieve this research objective, the following research questions were formulated: 1. "What is the basic concept of compassion fatigue in helping professions?" 2. "What are the predictor factors that can affect compassion fatigue in helping professions?" 3. "How is the development of compassion fatigue research in helping professions?".

#### Stage 2. Identifying relevant studies

**Fig. 1:**
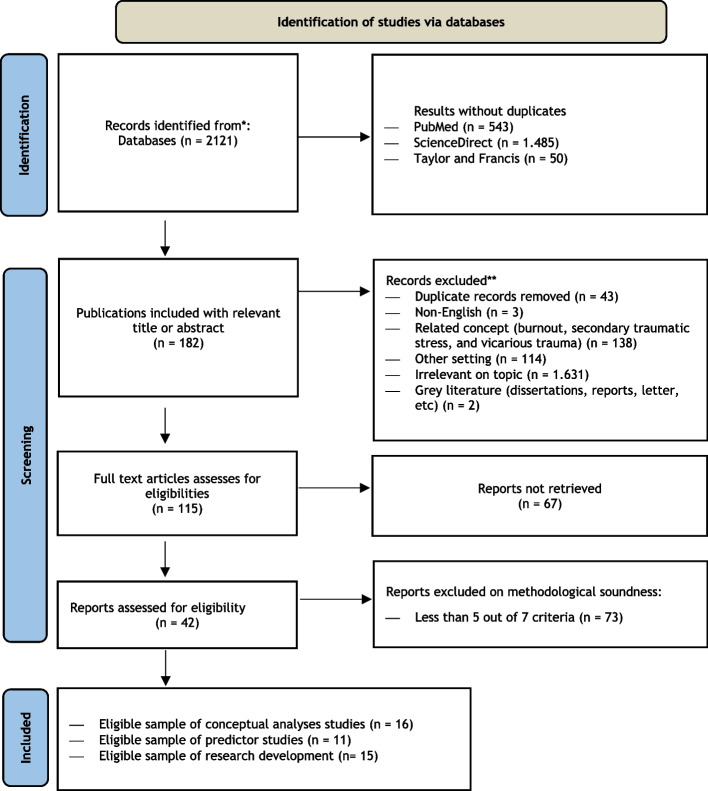
Flowchart review process. (Adaptation from PRISMA 2020 flow diagram)

In this study, a systematic literature search was conducted through electronic databases, namely ScienceDirect, PubMed, and Taylor and Francis. The search was conducted using Medical Subject Heading (MeSH) is a comprehensive controlled vocabulary for the puposes of indexing journal articles and book in the life sciences. including compassion fatigue, key terms: Compassion fatique (CF), beyond CF, understanding CF, Helping CF, Needing CF, Predictors CF, Compassion satisfaction, development CF, Effect CF, Prevalence CF, Factor CF, Solution CF, Method CF.. The Boolean indicator 'AND' was also used to narrow the literature search regarding concept analysis, predictor factors, and research developments on compassion fatigue in the context of healthcare, education, psychology, and social workers. The inclusion criteria were: 1. All research designs (qualitative, quantitative, mixed-method, and others); 2. The existence of an abstract; 3. Full text and accessible; 4. The research setting is health, education, psychology, and social work; 5. There are no restrictions on the year of publication; 6. Discuss the concept, model, or theory of compassion fatigue; 7. Discuss the predictors of compassion fatigue; 8. Discuss the development of compassion fatigue research. The exclusion criteria set are: 1. Multiple references; 2. Not using English; 3. Research in other settings; 4. Articles related to the concepts based on criteria of Burnout, Secondary Traumatic Stress, and Vicarious Trauma; 5. Not relevant to the topic; 6. Grey literature (dissertations, reports, letters, etc.). The Eligibility criteria are presented in Table 1 and Fig. 1 Flowchart review process as follows.

**Table 1:** Eligibility criteria

	**Quality Criterion**	Yes/No
1	Clear research questions and objectives	
2	A definition of measured concept(s)	
3	Valid and reliable measuring instrument	
4	Method description in detail	
5	Information on the size and type of the target population	
6	Research results and suggestions for further research	
7	Non literature review for factor predictor context	

(Source Adaptation from PRISMA 2020 flow diagram)

#### Stage 3. Selecting relevant studies

In selecting relevant literature, this research uses the PRISMA-ScR (Preferred Reporting Items for Systematic Reviews and Meta-Analyses) The stages are complete and detailed to conduct a literature review, there are 5 stages used, namely defining eligibility criteria, defining sources of information, literature selection, data collection and data item selection. 2020 flow diagram method which has four flow diagram phases [26]*.* After removing duplicate literature, the titles and abstracts of all articles were screened by the researchers by identifying exclusion criteria. After that, full text and non-full text were screened. Until the end of the selection stage, the final number of articles that meet the inclusion criteria and eligibility criteria is obtained. The eligibility criteria that must be met by each article are at least 4 of the 6 criteria. For criterion number 3, the star does not apply to articles that discuss the analysis of concepts, models, or theories.

#### Stage 4. Mapping the data

Data extraction from the selected literature was done using tables. Data extraction components for conceptualizing compassion fatigue include author name, year of publication, title, study type, profession, and purpose. The data extraction component for predictors of compassion fatigue includes the author's name, year of publication, study location, sample, research design, instruments used, and results. The data extraction component for compassion fatigue research development includes the author's name and year of publication, study location, research method, research population, instruments used, and results. All references were managed using Mendeley Desktop.

#### Stage 5. Synthesize data

The data synthesis stage in this research uses a narrative review approach, which focuses on collecting relevant information that provides context and substance to the author's overall argument [27]. Several characteristics of the studies that had been identified were then collected and summarized. The studies were summarized into a table and analysis was conducted on the tabulated data. Then the content was translated into the main themes, namely conceptualization, predictor factors, and research developments. The findings in this study were then interpreted, compared, and analyzed comprehensively.

The review process as illustrated in Fig. 1, started with 2,121 references retrieved from an electronic database. From the removal of multiple references (*n* = 43), not in English (*n* = 3), documents related to similar concepts such as Burnout, Secondary Traumatic Stress, and Vicarious Trauma (*n* = 138), research in other settings (*n* = 114), not relevant to the topic (*n* = 1,639), and grey literature (dissertations, reports, letters, etc.) (*n* = 2), 182 relevant articles were generated after the first stage of selection. After the first selection stage, the second selection stage was carried out by considering the availability of full text and accessible articles. A total of 182 full-text articles were included in the selection stage by considering the eligibility criteria. The final results showed that 67 articles were excluded and 42 articles met the eligibility criteria to be reviewed in this Scoping Literature Review. Validity and reliability are examined based on the credibility test using a triangulation strategy to check data on the same source with different techniques, then research sources from various reputable journals collected as many as 2121 which are then extracted with Vosviewer analysis to ensure they have accurate links. Transferability test, dependability test to audit the research results that replicate the research process and testing using ICR (intecoder reliability). besides the confirmability test.

### Findings

A total of 42 articles met the inclusion criteria and eligibility criteria to be reviewed in this Scoping Literature Review. This research will answer the research questions regarding of the discussion is divided into three, namely: 1) Conceptualization of Compassion Fatigue; 2) Predictor Factors of Compassion Fatigue; and 3) Development of Compassion Fatigue Research.

#### Conceptualization of compassion fatigue

The following describes the development of the idea of compassion fatigue philosophically from 1992 to 2019. In earlier informal research, Figley [3] argues that the term compassion fatigue was first introduced by Joinson in 1992 to explain the 'loss of the ability to nurture', namely the loss of nurturing ability in emergency nurses. The construct of compassion fatigue was not defined in depth by Joinson, but later the concept of compassion fatigue was adopted by Figley [28]. Figley [3] mentioned the phenomenon of the "cost of caring" which is the loss of self-perception (sense of self) towards the client being served because of listening to the client's story of fear, pain, and suffering so as to feel the same feelings that arise because of caring. The state of stress that arises from this condition is a result of the desire to help people who have experienced traumatic events.

Figley describes compassion fatigue as "behaviors and emotions that result from knowing about traumatizing events" [3]. Pioneered the development of compassion fatigue. The compassion fatigue model by [3, 14] It links empathy with the caregiver's ability to connect with and help clients. This model is a multifactor model, which is based on 10 variables to predict the onset of compassion fatigue in psychotherapists. The eleven variables include: 1. empathic ability; 2. empathic concern; 3. exposure to the client; 4. empathic response; 5. compassion stress; 6. sense of achievement; 7. disengagement; 8. prolonged exposure; 9. traumatic recollections; 10. life disruption.

In concept, Figley [3] suggests that helping professions that are vulnerable to compassion fatigue are therapists who are accustomed to working with traumatized people. There are four reasons why trauma workers are particularly vulnerable to compassion fatigue, including: 1. Empathy is a key strength for trauma workers to help traumatized people. The process of empathy can help understand what the traumatized person is going through, but in the process, empathy can traumatize trauma workers. 2. Most trauma workers have experienced traumatic events in their lives. This can be a danger for trauma workers to over-generalize about their experiences and over-provide methods or ways of coping to clients. 3. Traumatic events recounted by clients can trigger the re-emergence of past traumatic events that have not been resolved by trauma workers. 4. Traumatic events experienced by children can be profocative for the therapist. In general, compassion fatigue occurs in individuals who have demanding jobs, such as informal caregivers who are at risk when caring for family members with dementia [29].

Another compassion fatigue model was developed by Gentry and Baranowsky [30], the Accelerated Recovery Program, a model for understanding the various causes of compassion fatigue. There are several symptoms of compassion fatigue which are divided into three, namely: 1. Intrunsive Symptoms, such as disruption of personal activities by work-related problems, thoughts related to the client's traumatic experiences. 2. Avoidance Symptoms, such as loss of energy, loss of enjoyment in activities, avoiding to hear about the client's traumatic event (silence response). 3. Arousal Symptoms, such as increased anxiety, sleep disturbances, difficulty concentrating, changes in weight/appetite, and others.

A concept analysis was developed by Coetzee & Klopper [28] which defines compassion fatigue in nursing practice. The process of defining compassion fatigue is described into various categories, namely as risk factors, causes, processes, and manifestations. "Risks" are things that create the opportunity or possibility of developing compassion fatigue, such as contact with patients, self-use, and stress that is long-term, intense, and continuous. "Causes" are things that cause compassion fatigue, such as compassion discomfort and compassion stress. The process category describes the sequence of events or sequences of compassion fatigue, while the manifestation category involves the consequences of compassion fatigue and describes the physical, social, emotional, spiritual, and intellectual effects of compassion fatigue. Thus, the definition of compassion fatigue is the end result of a progressive and cumulative process that develops from compassion stress after a period of compassion discomfort caused by prolonged, continuous, and intense contact with patients, self-use, and exposure to stress. It develops from an uncomfortable state of compassion, which if not relieved through adequate rest, will lead to compassion stress that exceeds the nurse's endurance level and ultimately results in compassion fatigue.

Compassion fatigue is medically defined as "cynicism, emotional exhaustion, or selfishness", also defined as "fatigue, emotional distress, or apathy that results from the constant demands of caring for others or from the constant appeals of charitable organizations" [31]. The relationships that occur between individuals experiencing compassion fatigue are based on empathy and have the potential to produce profound responses (physical, psychological, spiritual, and social fatigue) [32]. Figley states that specific consequences as a result of compassion fatigue include sleep disturbances, fear, anxiety, difficulty concentrating, physical sensations such as tense muscles, feeling overwhelmed, tired and overwhelmed with hopelessness and isolation that result.

[12] proposes a transactional compassion fatigue model in physicians, where a physician will behave compassionately in a given situation determined by the dynamic interaction between the physician, patient and family, clinical situation and environmental factors. Physician factors that can influence a physician's compassionate response include gender, personality, basic traits or dispositions, past clinical experience, and communication skills. Patient and family factors include personality, gratitude, compliance and expectations of care. Clinical factors include the extent to which a physician consciously or unconsciously holds the patient "responsible", for their condition, the complexity of the situation and the physician's expertise. Environmental and institutional factors relate to job demands and feelings of control at work [12]. Suggests that it is the interaction between these factors that drives affection or produces inhibition. In detail, the analysis of the summary of included literature of compassion fatigue conceptual analyses by year of publication is given in Table 2.

**Table 2:** Summary of included literature of compassion fatigue conceptual analyses by year of publication

Author(s)	Year	Tittle	Type of Study	Profession	Aim
Figley, C	1995	Compassion Fatigue as Secondary Traumatic Stress Disorder	Literature review	Psychology	To review the scientific and clinical literature and to propose new approaches to conceptualizing, researching, treating traumatic stress
Figley, C	2002	Compassion Fatigue: Psychotherapists’ Chronic Lack of Self Care	Case study	Social work, psychotherapists	To discuss the concept, forms of compassion fatigue, among psychotherapists and compare it with simple burnout and countertransference
Gentry, JE	2002	Compassion Fatigue: A Crucible of Transformation	Empirical literature	Psychology practitioner	To explore the potential causes, prevention, and treatment of compassion fatigue and the damaging effects of helping those who have experienced trauma
Coetzee, S & Klopper, HC	2010	Compassion Fatigue within Nursing Practice: A Concept Analysis	Concept analysis	Clinical psychology	To define compassion fatigue in nursing practice and reveal deeper knowledge about compassion fatigue by identifying categories and characteristics, constructing theoretical and operational definitions, developing a model, and identifying empirical indicators
Day, JR & Anderson, RA	2011	Compassion Fatigue: An Application of the Concept to Informal Caregivers of Family Members with Dementia	Literature review	Clinical, Health care	To identify common themes across the literature as well as existing models of compassion fatigue in informal caregivers for family members with dementia
Lynch, SH & Lobo, ML	2012	Compassion Fatigue in Family Caregivers: A Wilsonian Concept Analysis	Literature review	Humanities, health care, psychology	To analyze the concept of compassion fatigue in family caregivers and their intense experiences, and identify the causes of family caregivers experiencing compassion fatigue
Fernando, AT & Consedine, NS	2013	Beyond Compassion Fatigue: The Transactional Model of Physician Compassion	Literature review on transactional model output	Psychological medicine	To present the Transactional Model of Physician Compassion, critique the utility of the concept of compassion fatigue, identify specific aspects of physicians' intrapersonal, interpersonal, clinical, and professional functioning that may impair or enhance compassion
Ledoux, K	2015	Understanding Compassion Fatigue: Understanding Compassion	Literature review	Clinical (nursing)	To discuss the construct of compassion fatigue in nursing, the nature, prevalence, and contribution of compassion in nursing, and the impact when compassion is absent or thwarted
Sheppard, K	2014	Compassion Fatigue among Registered Nurses: Connecting Theory and Research	Conceptual model and qualitative studies	Clinical (nursing)	To explain the challenges of applying one of the conceptual models widely used in research among nurses at risk of compassion fatigue
Sinclair, S, Raffin-Bouchal, S, Venturato, L, Mijovic-Kondejewski, J, & Smith-MacDonald, L	2017	Compassion Fatigue: A Meta-Narrative Review of The Healthcare Literature	Systematic literature review	Psychology and healthcare	To critically examine the construct of compassion fatigue and determine compassion fatigue as an accurate depiction of work-related stress in healthcare providers
Nolte, AG, Downing, C, Temane, A, & Hastings‐Tolsma, M	2017	Compassion Fatigue in Nurses: A Metasynthesis	Meta-synthesis	Clinical psychology	To interpret the results of qualitative research focusing on compassion fatigue to distill common understandings that can then be applied to caregivers
Steinheiser, M	2018	Compassion Fatigue among Nurses in Skilled Nursing Facilities: Discoveries and Challenges of a Conceptual Model in Research	Theory connection and literature review	Clinical psychology	To provide further insight into the commonly used conceptual model of compassion fatigue based on the results of the researcher's phenomenological study with nurses working in Skilled Nursing Facilities (SNFs)
Cross, LA	2018	Compassion Fatigue in Palliative Care Nursing	Concept analysis	Clinical psychology	To define compassion fatigue in the context of palliative nursing, within the discipline of palliative care nursing to make it relevant for care givers who are routinely exposed to patients with compassion fatigue
Butts, MM, Lunt, DC, Freling, TL, & Gabriel, AS	2019	Helping One or Helping Many? A Theoretical Integration and Meta-analytic Review of The Compassion Fade Literature	Literature review and meta-analytic	Psychology	To conduct the meta-analysis on compassion fade, synthesized 41 studies (95 independent samples with a total sample size of 13,259) spanning nearly two decades
Andrews, H	2019	Needing Permission: The Experience of Self-care and Self-compassion in Nursing. A Constructivist Grounded Theory Study	Constructivist grounded theory and semi-structured interviews	National Health Service (NHS) Trusts within the United Kingdom (UK)	To explore nurses' experiences of self-care and self-compassion, and the relationship with providing compassionate care to patients
Cavanagh, N, Cockett, G, Heinrich, C, Doig, L, Fiest, K, Guichon, J R., … & Doig, C. J	2019	Compassion Fatigue in Healthcare Providers: A Systematic Review and Meta-Analysis	Systematic review and meta-analysis	Medicine	To review the prevalence of compassion fatigue among healthcare practitioners and its association with demographic variables using narrative synthesis and meta-analysis methods. common in subscales

#### Factors of compassion fatigue

Factors associated with compassion fatigue have been widely studied in various helping professions. These factors can be categorized as personal factors, which include professional and sociodemographic factors, and work-related factors [33]. Personal factors that influence compassion fatigue include resilience [34], burnout [9], moral courage [9]*,* emotion control [35], empathy, compassionate care*,* mindfulness*,* self-judgement [36]*,* compassion satisfaction [37]*,* emotional exhaustion, physical status, number of children [35], female gender, mental health service utilization [38], and self-defeating humour [11]. Professional factors that influence compassion fatigue include professional competence, work experience, working in secondary hospitals, and applying passive coping styles [36], professional efficacy, [35, 39]. Work-related factors that influence compassion fatigue include traumatic experiences, life disruptions [36], job satisfaction [36], the number of patients admitted, the number of beds in service facilities [40], fluctuations in daily testimonies of patients suffering from [10], type of practice, having a multidisciplinary team, and emotional support outside the workplace [38].

A total of 10 of the 11 literatures reviewed in this scoping literature review used a cross-sectional research design. A total of three studies located in Italy, the United States, and Saudi Arabian [9–11] makes burnout a factor related to compassion fatigue. Burnout has a positive influence on compassion fatigue. High levels of burnout showed more emotional displays on days when they repeatedly witnessed patient suffering. Another study regarding personal factors, namely resilience is negatively correlated with compassion fatigue, meaning that individuals who have high resilience will have low compassion fatigue [34, 41, 42]. Moral courage has a slight and direct, but negative influence on compassion fatigue [9].

Life disruptions and traumatic experiences significantly predicted compassion fatigue and burnout. Female gender, significant emotional decline, use of mental health services, were predictors of high compassion fatigue. Self-oriented empathy plays an important role in influencing compassion fatigue through mediating mindfulness and counselor self-efficacy. Compassion fatigue effects high levels of burnout when seeing patients suffer. Other predictors: prior history of a severe illness, perspective taking, compassionate care, employee engagement, mindfulness, self-judgement, and over-identification. Other sociodemographic factors included income, marital status, mental health, empathy, mindfulness, and religious activity. The most studied potential predictors were age, gender, working hours, and workload [33]. The results of the literature review found, there is no literature that discusses predictor factors in the education profession.

In detail, The results of the litelature are organised based on the fields of education, psychology and medical which can be seen in the research sample. the analysis of the Summary of included literature of compassion fatigue factor predictor by year of publication is given in Table 3.

**Table 3:** Summary of included literature of compassion fatigue factor predictor by year of publication

Author(s)	Country	Sample	Design	Measure	Result
Li, J. N., Jiang, X. M., Zheng, Q. X., Lin, F., Chen, X. Q., Pan, Y. Q., … & Huang, L. (2023) [34]	China	307 intern nursing and midwifery students	Cross-sectional	Social Support Rating Scale, Connor-Davidson Resilience Scale, Chinese version of the Compassion Fatigue Short Scale	− Resilience is negatively correlated with compassion fatigue − Resilience is positively correlated with social support and subjective support, weakly correlated with objective support and usage of support − Compassion fatigue is negatively correlated with social support and subjective support − No significant relationship between compassion fatigue and objective support
Alshammari, M & Alboliteeh, M (2023) [9]	Saudi Arabian	684 for four government hospitals in Saudi Arabia	Cross-sectional	Nurses' Moral Courage Scale, Nurse Professional Competence Scale-Short Form, Maslach Burnout Inventory, and Nurses Compassion Fatigue Inventory	− Burnout has a positive influence on compassion fatigue − Professional competence has a direct influence and negative effect on compassion fatigue − Moral courage has a slight and direct, but negative effect on compassion fatigue − Moral courage significantly mediates the indirect effects of burnout and professional competence on compassion fatigue
Wang, J., Su, M., Chang, W., Hu, Y., Ma, Y., Tang, P., & Sun, J. (2023) [35]	United States	311 nurses	Cross-sectional	Chinese version of the Compassion Fatigue Scale, the Maslach Burnout Inventory General Survey (MBI-GS), Emotional Labour Scale, the Social Support Rate Scale (SSRS)	− Physical status, number of children, emotional control, professional efficacy, emotional exhaustion can affect compassion fatigue − Cynicism, social support, work experience, work status and night shift are predictors of compassion statisfaction
Hairong Yu, Anhua Qiao, Li Gui (2016) [39]	China	669 oncology nurses	Cross-sectional	Chinese version of the Professional Quality of Life Scale for Nurses, the Chinese version of the Jefferson Scales of Empathy, the Simplified Coping Style Questionnaire, the Perceived Social Support Scale, and the Chinese Big Five Personality Inventory brief version	Longer experience working in nursing, working in secondary hospitals, and applying passive coping styles affect high compassion fatigue in oncology nurses
Jalal, A, Debra, J, Kim, U (2020) [40]	Australia	516 critical care nurses	Cross-sectional	Professional Quality of Life Scale, version 5 (ProQol-5)	− The number of patients treated and the number of beds in the service facility affect the level of nomination of sensitive indicators of nurses − Resilience level is a predictor of compassion fatigue
Craigie, M., Osseiran-Moisson, R., Hemsworth, D., Aoun, S., Francis, K., Brown, J., … Rees, C. (2016) [37]	United States	273 nurses from 1 metropolitan tertiary acute hospital in Western Australia	Cross-sectional	Professional Quality of Life Scale Depression Anxiety Stress Scale, and the State-Trait Anxiety Inventor	− Trait-negative affect is an important factor contributing to compassion fatigue and secondary traumatic stress − Compassion satisfaction is an important source of internal factors that protect against burnout, but has an indirect effect on protecting against secondary traumatic stress
Hairong Yu, Anhua Qiao, Li Gui (2021) [36]	China	186 emergency nurses	Cross-sectional	Chinese version of the Jefferson Scale of Empathy for Nurses, Chinese version of the Self-compassion Scale, Chinese version of the Work-Related Quality of Life Scale, Professional Quality of Life Scale	− Empathy, job satisfaction, and self-compassion explain differences in compassion fatigue, burnout, and compassion satisfaction − Life disruptions and traumatic experiences significantly predict compassion fatigue and burnout − Other predictors: prior history of a severe illness, perspective taking, compassionate care, employee engagement, mindfulness, self-judgement, and over-identification
Mackenzi, N, Cassandra Pruitt, Alex Sarosi, Jill Berkin, Joanne Stone, Andrea S. Weintraub (2023) [38]	United States	366 White physicians working in academic medical centers	Cross-sectional	Compassion Fatigue and Satisfaction Self-Test and a questionnaire of professional and personal characteristics	− Female gender, self-report of significant emotional decline, use of mental health services, were predictors of high compassion fatigue − Compassion fatigue, compassion satisfaction, and burnout were associated with various risk factors, such as practice type, having a multidisciplinary team, emotional support outside the workplace
Zhang, L, Ren, Z, Jiang, G, et al. (2020) [65]	China	712 hotline psychological counselors	Cross-sectional	Professional Quality of Life Scale (ProQoL), version 5, Interpersonal Reactivity Index-Chinese Version (IRI-C), Mindful Attention Awareness Scale-Chinese version (MAAS-C), Chinese version of the Counselor Self-Efficacy Scale (CSES-C)	Self-oriented empathy plays an important role in influencing CF through mediating mindfulness and counselor self-efficacy
Timofeiov-Tudose, I Măirean, C (2023) [11]	United States	189 medical staffs	Cross-sectional	The Compassion Scale, Work-related Humour Styles Questionnaire, The Professional Quality of Life Scale	− Self-enhancing and affiliative humour were positively related, while self-defeating humour was negatively related to compassion satisfaction − Burnout and secondary traumatic stress were negatively related to self-enhancing humour and positively related to self-defeating humour − Compassion moderates the relationship between affiliative humour and secondary traumatic stress
Portoghese, I, Galletta, M, Larkin, P, et al. (2020)	Italia	39 hospice professionals from two Italian hospices	Diary research	Professional Quality of Life Assessment R-IV Scale (ProQOLRIV)	− Fluctuations in daily testimony of suffering patients were positively associated with daily use of positive emotional regulation − High burnout levels showed more emotional displays on days when they repeatedly witnessed patient suffering − Compaasion fatigue exerts an effect on high burnout levels when seeing patients suffer

#### Examination in various professional fields and the global development of compassion fatigue research

The compassion fatique starting from the research methods used, research samples, so that further research will know the follow-up process to fill the research gaps based on research methods and samples or strengthen the study of research methods. The development of compassion fatigue research is found in literature from various countries, such as the United States, China, Portugal, Canada, Iran, and England. Compassion fatigue has been studied by various professional fields, including healthcare [15–19, 22, 43–48]*,* education [20, 49], psychology [50], and social workers [14]. The research methods used in the compassion fatigue literature found, namely using quantitative and qualitative approaches with various research designs. Some studies that use the literature review method include [2, 12, 13, 28–32, 51–56].

Compassion fatigue research is conducted on various helping professions, ranging from nurses, teachers, doctors, psychologists, coaches, paramedics, psychotherapists, pastors, and police. Research on compassion fatigue in the nursing profession is divided into several contexts, namely the context of prevalence with descriptive methods and cross-sectional survey designs [1, 22, 46], context of intervention with experimental method [44, 45], context of correlation study with descriptive correlation method [18, 47, 57]. There are differences in prevalence rates among nurses based on gender, age, education level, changes in nursing management, and system changes [17, 47]. Compassion statisfaction, compassion fatigue, and hardiness did not change during COVID-19 compared to before the COVID-19 period [22]. A study on interventions for oncology nurses found that mindfulness-based interventions (MBIs) mediated changes in burnout, anxiety and stress, and life satisfaction [58]. Developing sharper self-awareness is essential for recognizing and reducing CF and burnout [45]. A correlational study of mental health professionals and medical social workers found mindfulness to be a protective factor against compassion fatigue regardless of professional or student status [18]. Another study found that poor sleep quality, low job satisfaction, more working hours, and exposure to cigarette smoke were associated with compassion fatigue [57].

Research on compassion fatigue in the education profession found that educators with longer tenure in education and educators of color reported higher levels of compassion fatigue than their peers [49]. The importance of promoting school connectedness with educators in an interactive way to improve educators' occupational well-being. There were significant differences in compassion fatigue levels across helping professions. The highest levels reported were doctors, educators, home caregivers, nurses and psychologists. The lowest CF levels were psychotherapists and trainers which could be due to the poor quality of health and education systems in Central Europe [1]. In detail, the analysis of the Summary of included literature of research on compassion fatigue by year of publication is given in Table 4.

**Table 4:** Summary of included literature of research on compassion fatigue by year of publication

Author(s)	Country	Method	Population	Measure	Result	Conclusion
Healthcare						
Potter, P Deshields, T Divanbeigi, J, Julie, C Norris, L Olsen, S (2010) [15]	United States	Descriptive, cross-sectional survey	153 healthcare providers included RNs, medical assistants, and radiology technicians	ProQOL R-IV	Average compassion statisfaction 38.3 (SD = 7.2) Average burnout 21.5 (SD = 6.4) Average compassion fatigue 15.2 (SD = 6.6)	Staff working in the inpatient nursing unit had the highest percentage of compassion fatigue risk scores
Severn, M. S., Searchfield, G. D., & Huggard, P. (2011) [16]	United States	Descriptive, cross-sectional postal survey	82 audiology practitioners that were full members of the New Zealand Audiological Society (NZAS)	AOSQ and ProQOL	Six stressors dominate clinical audiology: (1) time demands, (2) audiology management, (3) patient contact, (4) clinical protocols, (5) patient accountability, and (6) administration or equipment. There was a significant relationship between increasing audiologist age and the risk of burnout. Stress due to patient contact was the strongest predictor of compassion fatigue	This study identified sources of stress for clinical audiologists and various factors that contribute to professional quality of life
Sacco, T, Ciurzynski, M, Harvey, M Ingersoll, G (2015) [17]	United States	Quantitative, descriptive, cross-sectional design	221 adult, pediatric, and neonatal critical care nurses	Professional Quality of Life (ProQOL)	There were significant differences in compassion statisfaction and compassion fatigue based on gender, age, education level, unit, acuity, nursing management changes, and major system changes	Understanding the elements of professional quality of life can have a positive effect on the work environment. The relationship between professional quality of life and healthy work environment standards requires further investigation to develop appropriate interventions
Duarte, J & Pinto-Gouveia, J (2017) [21]	Portugal	Quantitative, descriptive, cross-sectional design	298 registered nurses from public hospitals	Professional Quality of Life (ProQOL)-V	Correlation analysis showed that empathy-based guilt was positively associated with empathy, burnout, and compassion fatigue. When empathy was associated with empathy-based guilt, this led to greater levels of burnout and compassion fatigue	Nurses who experience pathogenic feelings of guilt may have compromised their well-being, and this should be addressed in training programs that aim to prevent or treat burnout and compassion fatigue
Tucker, T., Bouvette, M., Daly, S., & Grassau, P. (2017) [45]	Canada	Quantitative, experimental	165 third year trainees at a Canadian Medical School	Professional Quality of Life (ProQOL)	The results highlight the importance of 1) Recognizing Signs & Symptoms of Individual Stress, CF and Burnout; 2) Normalizing Stress, CF and Burnout in Students and Clinicians; 3) Learning to Manage Stress on Your Own. There was a decrease in compassion statisfaction and an increase in burnout between the beginning and end of the third year	Sharper self-awareness is essential for recognizing and reducing CF and burnout, and the third-year medical curriculum seems to be a good start
Duarte, Joana Pinto-Gouveia, José (2017) [44]	Portugal	Quantitative, experimental	94 oncology nurses	Professional Quality of Life (ProQOL)-V	Changes in mindfulness mediated changes in burnout, anxiety and stress, and life satisfaction; changes in self-compassion mediated the impact of the intervention on burnout, depression, anxiety, stress and life satisfaction; and psychological inflexibility mediated reductions in burnout, compassion fatigue, depression and stress	These findings contribute to a growing body of research examining the underlying mechanisms at work in MBI, and highlight the importance of mindfulness, self-compassion and psychological inflexibility as key change processes
Brown, J, Ong, J, Mathers, J, &Decker, J (2017) [18]	United States	Quantitative, exploratory study	mental health professionals (*n* = 40) and Medical Social Workers students (*n* = 111)	Five Facet Mindfulness Questionnaire (FFMQ) dan Professional Quality of Life (ProQOL)	There was a moderate negative correlation between compassion fatigue and mindfulness, with high levels of compassion fatigue associated with lower levels of mindfulness. There were no statistically significant differences between mental health workers and MSW students on the combined dependent variables	Mindfulness protects against compassion fatigue regardless of professional or student status
Roney, L & Acri, M (2018) [47]	United States	Quantitative, descriptive correlational	318 members of the Society of Pediatric Nurses	Job Satisfaction Survey (JSS) and the Professional Quality of Life (ProQOL)	More than three-quarters (245, 76%) of the sample had another career before becoming a nurse. The mean job satisfaction level of the sample was 149.8 (SD = 29.74), which was significantly higher than the mean reported for nurses. Bivariate analysis revealed a significant relationship between gender and compassion statisfaction, with women more likely than men to demonstrate compassion statisfaction (t = 1.967, *p* = 0.05, df = 298)	The majority of nurses had high levels of compassion statisfaction and job satisfaction; furthermore, female gender was associated with higher levels of compassion statisfaction
Wang, J., Okoli, C., He, H., Feng, F., Li, J., Zhuang, L (2020) [19]	China	Quantitative, descriptive, cross-sectional survey	1044 registered nurses from different nursing departments	Professional Quality of Life (ProQOL)	The average number of hours worked per day was a positive factor for burnout, while being married/having an unmarried partner, job satisfaction, hours of sleep per day, and sleep quality were negative factors for burnout. Poor sleep quality, low job satisfaction, more working hours, and exposure to cigarette smoke were associated with secondary traumatic stress	Revealed the serious phenomenon of poor professional quality of life among Chinese nurses. Findings may provide clues to help nursing managers identify nurses' vulnerability to compassion fatigue burnout and implement targeted strategies to reduce nurses' burnout and secondary traumatic stress
Pehlivan, T & Güner, P (2020) [48]	England	Quantitative experimental	125 oncology-haematology nurses	Perceived Stress Scale	The results of the multilevel model analysis showed no statistically significant differences between the mean scores of compassion fatigue, burnout, perceived stress, and resilience of nurses in the short-term or long-term groups or in the control group	Short-term programs are preferred to encourage more participation among nurses. Further studies that include environmental improvements as well as training programs are needed
Zakeri, M, Rahiminezhad, E, Salehi, F, Ganjeh, H Dehghan, M (2021) [22]	Iran	Quantitative, descriptive, cross-sectional survey	508 clinical nurses from one public hospital in southern Iran	Professional Quality of Life (ProQOL)	Compassion statisfaction, compassion fatigue, and hardiness scores were not significantly different during the COVID-19 period compared to before the COVID-19 period (*p* > 0.05)	Compassion statisfaction, compassion fatigue, and hardiness did not change during COVID-19 compared to before the COVID-19 period. During COVID-19, hardiness became a predictor of compassion statisfaction and compassion fatigue. Compassion statisfaction can be increased and compassion fatigue can be reduced by strengthening hardiness in nurses
Educator						
Yang, C Manchanda, S Greenstein, J (2021) [49]	United States	Quantitative, empirical research	321 educators recruited from a large urban district in Northern California	Professional Quality of Life (ProQOL); Online Teaching Self-Efficacy Scale (DTSES); Distance Learning School Connectedness Scale (DLSCS)	Educators with longer tenure in education and white educators reported higher levels of compassion fatigue than their peers. White educators also reported lower levels of online teaching self-efficacy than their peers	The importance of promoting school connectedness with educators interactively in improving educators' work well-being and influencing compassion fatigue and online teaching self-efficacy
Chen, F., Ge, Y., Xu, W., Yu, J., Zhang, Y., Xu, X., & Zhang, S. (2023) [20]	China	Quantitative, descriptive, cross-sectional survey	1049 kindergarten teachers	Kindergarten Teachers’ Mindsets Toward Children; Motivation for Teacher Empathy (MTE); Compassion Fatigue Short Scale (C-CF Short Scale)	Teacher empathy motivation mediates the negative relationship between kindergarten teachers' mindset towards children and compassion fatigue. Job stress and social support moderate the relationship between kindergarten teachers' mindset towards children and teacher empathy motivation	The proposed moderated mediation model was found to be valid. In addition, the findings of this study have practical implications for developing evidence-based interventions to address kindergarten teachers' compassion fatigue
Psychology						
Craig, C.D, & Sprang, G (2010) [50]	England	Quantitative, experiment	1000 clinical psychology and 1000 clinical social work were selected from the 2003 National Association of Social Worker’s	ProQOL-III	46% scored above the cut-off for compassion satisfaction by half of the clinicians 5% of the clinicians scored above the cut-off for burnout 5% scored above the cut-off for compassion fatigue	Utilization of evidence-based practices predicted a statistically significant decrease in compassion fatigue and burnout, and increased compassion satisfaction
Mixed Helping Professionals						
Ondrejková, N Halamová, J (2022) [1]	England	Quantitative, descriptive, cross-sectional survey	102 nurses, 44 doctors, 57 paramedics, 39 home nurses, 66 teachers, 103 psychologists, 40 psychotherapists and coaches, 76 social workers, 39 priests and pastors and 41 police officers	Professional Quality of Life (ProQOL)-V; Sussex-Oxford Compassion for the Self Scale (SOCS-S)	There were significant differences in the level of compassion fatigue in various helping professions. The highest levels reported were doctors, educators, home caregivers, nurses and psychologists. The lowest levels of CF were psychotherapists and coaches	High CF rates among healthcare professionals (doctors, nurses, and psychologists) and educators may be due to the poor quality of health and education systems in Central Europe

## Discussion

This scoping literature review discusses the concept, predictors, and development of compassion fatigue research in various helping professions. This study reviewed 42 articles that met the inclusion criteria and eligibility criteria. A total of 16 articles were sampled in the discussion of conceptual analysis, 11 articles were sampled in the discussion of predictor factors, and 15 articles were sampled in the discussion of research developments on compassion fatigue.

Most of the literature reviewed in this study revealed that the term compassion fatigue was first introduced by Joinson in 1992 to explain the 'loss of the ability to nurture' in emergency nurses. Nurses feel tired, depressed, angry, ineffective, apathetic and uncaring, and experience somatic complaints such as headaches, insomnia, and indigestion due to heavy workloads and complex patient needs and these responses increase over time as a result of cumulative stress. Theoretically, Joinson argues that the term compassion fatigue is synonymous with the term burnout. Joinson (1992) revealed that intense stress can dominate an individual and interfere with his or her ability to function which makes the individual angry, ineffective, apathetic, and depressed. The symptoms exhibited by individuals in this condition are classified as burnout symptoms, especially if they occur on the job [59]. Explains that elements of burnout can occur in any environment, but there is one unique form of burnout, called compassion fatigue that affects people in the nursing profession. Unlike other burnouts, compassion fatigue is directly linked to specific people: nurses, ministers, counselors, and other caregiving professions. However, the nursing profession is the most vulnerable to compassion fatigue. There are four reasons why individuals should be aware of compassion fatigue and respond immediately, including: 1) Compassion fatigue is emotionally devastating; 2) Leads to the caregiver's personality; 3) External resources that cause it cannot be avoided; 4) Compassion fatigue is almost difficult to detect without a high level of awareness [59]. The three main issues of compassion fatigue in caregivers include: 1) Caregivers may perform some concrete functions, most of which are performed by themselves. They have to renew and rebuild themselves; 2) Human needs are infinite. Caregivers tend to feel that they can always provide help, but actually they cannot; 3) Caregivers perform multiple roles which can cause mental conflict [59]. Explains that nurses are highly valued for putting the needs of other individuals above their own. As they reach more advanced stages in their lives, they become more distant in their sensitivity to stressors as they learn how to stop them. Psychologically, nurses are prone to compassion fatigue. Symptoms of compassion fatigue follow the usual pattern of stress, recognizable symptoms include: 1) Becoming forgetful or having a short attention span; 2) Feeling tired and having periodic stomach aches and headaches; 3) Low resistance and frequent illness [59]. Being aware of symptoms and responding quickly and taking time to assess emotional health is key in dealing with compassion fatigue [59]. Other suggestions for dealing with stress realistically include: 1) Do not feel responsible for solving problems that are not part of the task and do not take over the problem; 2) Give yourself spiritual nourishment. Stay humble about what can and cannot be done [59]. Steps that can be taken for individuals who are trying to get out of compassion fatigue and become effective caregivers include: 1) Try to learn about boundaries; 2) Use humor to emphasize boundaries to others; 3) Give yourself permission to have personal time; 4) Give balance in life and set priorities; 5) Think about self-image; 6) Build a spiritual side (Chase in [59]). Reflect, assess, and renew oneself regularly so that individuals will be emotionally healthy [59].

There is literature that first discusses compassion fatigue, namely by [60] and [61]. [60] discussed her personal experience of feeling that her colleague was affected by compassion fatigue [60]. Is a doctor who has a serious illness that he has been suffering from for 5 years and cannot be told to other colleagues. However, he felt very disappointed because in reality the person who was considered capable of being relied on was the one who did not expect to hear how serious it was for him. This then raises the question that perhaps the biggest reason for the high suicide rate among doctors is because they are afraid to share their difficulties with their colleagues. Other literature by [61] discussed the national conference by Edward Poliandro, PhD, on stress and burnout in healthcare social workers. Increased compassion fatigue in caregivers is caused by the increased volume and frequency of problems, such as drugs, alcohol, violence, that caregivers face every day. Compassion fatigue is especially likely to occur in healthcare because helping professions, who are naturally compassionate people, often put the needs of others first and their own needs last. Putting one's own needs last over a period of time will lead to compassion fatigue and burnout (Poliandro in [61]. Compassion fatigue and burnout are different stages in a series of unity. Compassion fatigue occurs before entering the burnout stage [61]. Defines compassion fatigue as a psychological and emotional state, where the caregiver feels drained, numb, and exhausted. Unlike normal fatigue which can be alleviated by resting, compassion fatigue is chronic. Individuals lack the energy to interact with friends and family and find it difficult to react emotionally to others. Other signs are cynicism, anger, hostility, and irritability which are protective barriers against feeling overwhelmed [61]. If left unattended, compassion fatigue will lead to deeper problems of burnout or clinical depression that are difficult to correct [61]. Burnout usually occurs when chronic stress is left unrecognized for a long time.

Conceptual analysis of compassion fatigue cannot be generalized across healthcare settings or professions [13]. It is difficult to imagine how a conceptual model of compassion fatigue could be equally relevant and applicable to a variety of helping professions, such as a psychotherapist who may be chronically burdened with distressing memories of her clients, a nurse who is experiencing acute depression, a family practitioner who has long been involved in the care of patients with chronic illnesses, and a physician who has expressed difficulty remaining compassionate despite not being exposed to trauma [12]. This is also what is then limited in this study. Second, the antecedents and pathways of compassion fatigue should be based on a conceptual model that specifies the various elements of compassion fatigue, so that their determinants and relationships with each other can be clearly delineated. Currently, conceptual analyses of compassion fatigue tend to focus on limited aspects of compassion (e.g., behaviors, motivators) rather than its overall construction [13]. Compassion is multifaceted, involving benevolence, proactive response, attempts to understand, relational communication, confrontation, and action. Until the risk factors, antecedents, pathways and manifestations of compassion fatigue are identified, based on a valid multifaceted compassion model that is consistent and relevant across multiple healthcare professions, the construct validity of compassion fatigue is increasingly questioned. Third, the presence of any of the more than forty physical, behavioral, psychological and spiritual symptoms can validate the diagnosis of compassion fatigue, although generally more than one symptom must be demonstrated before a healthcare provider is identified as experiencing compassion fatigue [62]*.*

A meta-narrative study states that 'compassion fatigue rests on the most fragile of foundations' [2]. This literature review identifies the concept of compassion fatigue as a euphemism for different types of occupational stress uniquely associated with healthcare providers that has no valid construct and therefore cannot be empirically validated or measured. As a result, researchers often equate compassion, sympathy, and empathy in conceptualizing compassion fatigue, ignoring the characteristics, motivators, outcomes, and responses that differentiate the two in the process. Conceptually, sympathy is understood as "a pity-based response to distressing situations characterized by a lack of relational understanding and self-preservation on the part of the respondent" [13]. Empathy has two components, namely cognitive empathy and affective empathy. Cognitive empathy is "the ability to understand another person's intentions, desires, beliefs resulting from reasoning (cognitively) about the other person's circumstances" and affective empathy is involving emotional resonance or feelings with the person in need. Although the concept of compassion fatigue is used synonymously with other work stress terms, an equally important issue is the unification of the etymological roots on which these are based.

The models developed by experts are aligned in terms of the positive and negative outcomes of caring for clients. The models are more vague in relation to the process or etiology of compassion fatigue, but the main aspect of concern is the balance of resources. [62] further builds on these models and explains the process or etiology of compassion fatigue through the application of conservation of resources (COR) theory to explain resource balance, and social neuroscience of empathy research to explain empathy use and stress appraisal, named the Compassion Fatigue Model.

To explain how resource balance affects the etiology of compassion fatigue or compassion statisfaction, COR theory is applied, as it is an integrated model of stress, and consists of several stress theories. The main principle of COR theory is that because people value resources, they seek to acquire resources they do not have, they defend resources they do have, they protect resources that are threatened, and they develop resources by ensuring that their resources can be put to their best use. Following this principle, COR theory includes two main principles and several secondary consequences. The first and most important principle is that "resource loss is much more important than resource gain." The second principle states that "people should invest resources to protect against resource loss, recover losses, and gain resources." These principles are implemented to show the balance of resources that influence the etiology of compassion fatigue or compassion statisfaction.

To explain how empathy and stress appraisal affect the etiology of compassion fatigue or compassion statisfaction, social neuroscience of empathy research. Empathy is the caregiver's ability to understand, imagine, or infer the client's suffering, grief, or pain, and express motivation to improve the patient's experience, with full awareness of the differences between him or herself and the patient. Social neuroscience has shown that certain neural structures, namely the bilateral anterior insular cortex and medial/anterior cingulate cortex, are associated with empathy for pain, and this overlaps with activation during directly experienced pain. In neuroscience it is also explained that there are two modes of information processing in empathy: experiential or propositional processing modes. The experiential processing mode is an involuntary response or bottom-up approach, whereas the propositional processing mode is usually a voluntary response or top-down approach.

The resources described in this Compassion Fatigue Model (CFM) theory include object, conditional, personal, and energy resources. Object resources are resources that have a physical presence and are valued based on their function or status, such as adequate infrastructure and staff. Conditional resources are structures or states that lay the foundation for access or ownership of other resources, such as spirituality or health. Personal resources are acquired through learning and result from role modeling, education and adoption. Personal resources include personal skills and traits. Personal skills include employability skills and leadership abilities, while personal traits include self-esteem and resilience [63]. Energy resources include actual energy, time, and knowledge [63]. This Compassion Fatigue Model (CFM) suggests that nurses with poor resources are more likely to experience compassion fatigue. While most resources relate to the individual level (conditional, personal, and energy resources), the practice environment (object resources) is an external resource that can be addressed through policies governing the healthcare sector and healthcare facilities and units. A positive practice environment results in better outcomes for nurses and patients [62].

This scoping literature review also sought to identify predictors of compassion statisfiction and compassion fatigue in various helping professions. A total of 16 articles on predictors reviewed in this study showed that most predictors of compassion fatigue were associated with burnout [9–11]. Studies that examine factors that predict compassion fatigue in this review are dominated by nurses, other professions are hospital staff and psychologist counselors. Factors that influence compassion fatigue in nurses and hospital staff are resilience, social support and subjective support, burnout, personal competence, moral courage, physical status, number of children, emotional control, professional efficacy, emotional exhaustion, cynicism, social support, work experience, work status, night shift, length of work experience, number of patients admitted, number of beds in the facility, trait-negative affect, empathy, job satisfaction, and self-compassion, life disruption, traumatic experience, prior history of a severe illness, perspective taking, compassionate care, employee engagement, mindfulness, self-judgement, and over-identification. Female gender was also a predictor of high compassion fatigue. In addition, fluctuations in daily interactions with suffering patients were also positively associated with the use of daily positive emotional regulation. For predictor factors that affect compassion fatigue in counselor psychologists, self-oriented empathy has an important role in influencing compassion fatigue through the mediation of mindfulness and counselor self-efficacy. Another factor is that sometimes burnout can start with colleagues. When people around feel tense, impatient, and rushed, individuals will also feel drawn to the same reaction (Joinson, 1992). Colleagues who feel tired, unsympathetic and cynical can sap the energy and enthusiasm of other individuals [59].

A discussion of the development of compassion fatigue research is also reviewed in this study. The development of compassion fatigue research is found in literature from various countries, such as the United States, China, Portugal, Canada, Iran, and England. Compassion fatigue has been studied by various professional fields, including healthcare [15–19, 22, 43–48]*,* education [20, 49], psychology [50], and social workers [14]. The research methods used in the compassion fatigue literature found, namely using quantitative and qualitative approaches with various research designs. Some studies that use the literature review method include [2, 12, 13, 28–32, 51–56]. Articles that use a cross sectional research design include [9–11, 34–38, 64, 65]*.*

The implications of the research results examine the conceptualisation of compassion fatique as a philosophical and theoretical basis developed to deeply understand the series of situations created from the process of research experience, meaning, generation of ideas, construction of thought flow and culture that have an impact on gaining and strengthening understanding of compassion fatique. Scoping the Compassion Fatigue literature has implications for providing comprehensive analyses of the impact of philosophical research, compassion fatigue, patterns of research methods to follow up or explore emerging research, and accurately locate research gaps for basic, applied, and developmental research. Scoping the Compassion Fatigue literature informs decision-making for policy makers related to curriculum, resources, and regulations. Scoping Compassion Fatigue literature becomes a foundational piece in creating a platform that builds on theoretical and empirical data analyses to support strategy design, implementation in educational settings.

## Conclusion

The concept of compassion fatigue developed by experts is still unclear in relation to the process or etiology of compassion fatigue. The various models developed cannot be generalized and applied relevantly to various helping professions. The models that have been developed are dominated by the healthcare field. Further analysis is needed to develop a conceptual analysis of compassion fatigue that focuses on other fields of work more specifically and comprehensively. The development of the compassion fatigue model as a conceptual analysis needs to include various elements or aspects of compassion fatigue, a clear depiction of the determining factors and their relationship with each other, as well as the validity and determination of the physical, behavioral, psychological and spiritual symptoms that affect it so that individuals who experience compassion fatigue can be identified appropriately. In the literature found, compassion fatigue is influenced by personal factors which include professional factors and sociodemographic factors as well as work-related factors. Research on compassion fatigue has developed a lot, especially in the health sector, especially nursing using experimental, cross-sectional, and literature review research methods. Expansion of research development is needed in the fields of education and psychology.

## Data Availability

Data is available from the corresponding author upon reasonable request.
